# Dissociation between subjective sleep quality and lipid dysregulation in underground miners: night shift work as an independent risk factor for hypercholesterolemia

**DOI:** 10.3389/fpubh.2026.1759787

**Published:** 2026-04-13

**Authors:** Xiaochuan Zhao, Wei Wang, Na Li, Yuanyuan Gao, Lulu Yu, Mei Song, Xueyi Wang, Lan Wang

**Affiliations:** Mental Health Center, The First Hospital of Hebei Medical University, Shijiazhuang, Hebei, China

**Keywords:** circadian rhythm, lipid metabolism, occupational health, shift work, sleep quality, total cholesterol

## Abstract

**Objective:**

Shift work disrupts circadian rhythms and is established as a risk factor for metabolic syndrome. While poor sleep quality is often hypothesized as the primary mediator linking shift work to dyslipidemia, the extent to which circadian misalignment affects lipid metabolism independently of sleep complaints remains unclear. This study aimed to investigate the independent and combined effects of shift work and sleep quality on serum lipid profiles in a cohort of male underground miners.

**Methods:**

A cross-sectional analysis was conducted on 921 male miners from the Kailuan Group. Participants were categorized by work schedule into Night Shift (0:00–7:59, *n* = 326) and Day/Morning Shift (8:00–23:59, *n* = 595) groups to ensure distinct circadian exposure profiles. Sleep quality was assessed using the Pittsburgh Sleep Quality Index (PSQI), with a score >5 defining poor sleep. Fasting serum lipids—Total Cholesterol (TC), Triglycerides (TG), High-Density Lipoprotein Cholesterol (HDL-C), and Low-Density Lipoprotein Cholesterol (LDL-C)—were quantified. General Linear Models (GLM) and interaction analyses were employed to assess associations, adjusting for age, education, smoking, and alcohol consumption.

**Results:**

Night shift workers exhibited significantly higher global PSQI scores compared to day workers (4.70 ± 3.06 vs. 3.59 ± 2.71, *P* < 0.001). While the overall prevalence of dyslipidemia did not significantly differ (32.52% vs. 27.57%, *P* = 0.114), night shift work was significantly associated with elevated mean TC levels (5.36 ± 2.83 vs. 5.09 ± 1.09 mmol/L, *P* = 0.039) independent of covariates and occupational factors. Surprisingly, subjective sleep quality (PSQI) showed no significant correlation with lipid parameters (All *P* > 0.05). In the stratified interaction analysis, the highest TC levels were observed in the “Night Shift + Good Sleep” subgroup (5.51 ± 3.43 mmol/L), significantly differing from the “Day Shift + Good Sleep” reference group (4.96 ± 1.06 mmol/L, *P* = 0.039).

**Conclusion:**

Night shift work is associated with elevated total cholesterol in male miners, an effect that persists even among those reporting good sleep quality. This suggests that circadian misalignment governs lipid dysregulation via pathways distinct from subjective sleep disruption. Occupational health interventions should target circadian phase management beyond basic sleep hygiene education.

## Introduction

The modernization of industrial operations has necessitated a global increase in non-standard work schedules, with approximately 17%−19% of the workforce in industrialized nations engaged in shift or night work (1, 2). In the mining sector, continuous operations are mandatory, exposing workers to chronic circadian disruption. A growing body of epidemiological evidence indicates that shift work is not merely a social inconvenience but a potent independent risk factor for metabolic diseases, including obesity, type 2 diabetes mellitus, and cardiovascular disease (CVD) (3–5). Dyslipidemia, particularly elevated total cholesterol (TC) and triglycerides (TG), represents a critical pathophysiological bridge connecting occupational stress to atherosclerotic outcomes (6–8).

The mechanisms underpinning shift work-induced dyslipidemia are complex. Traditionally, sleep disruption has been posited as the primary culprit. Shift work disorder (SWD), characterized by insomnia and excessive sleepiness, affects a significant proportion of night workers due to the misalignment between endogenous circadian rhythms and work schedules, with a systematic review reporting a pooled prevalence of 26.5% among shift workers (9). Sleep fragmentation and deprivation are known to disrupt appetite-regulating hormones (leptin and ghrelin), leading to increased caloric intake and subsequent lipid derangement; a 2020 meta-analysis confirmed that short sleep duration is associated with reduced leptin and elevated ghrelin levels (10). This hormonal imbalance contributes to dyslipidemia, as evidenced by a 2023 systematic review which highlighted that shift work, particularly permanent night shifts, promotes dyslipidaemia through circadian disruption and metabolic alterations (11). Consequently, many occupational health interventions focus almost exclusively on improving sleep hygiene, as reflected in recent non-pharmacological management guidelines that prioritize sleep hygiene education for shift workers (12).

However, recent chronobiological research suggests that sleep quality may not account for the entirety of the metabolic risk (13, 14). Molecular clocks located in peripheral tissues, particularly the liver and adipose tissue, tightly regulate lipid biosynthesis and transport. Experimental studies in humans have demonstrated that circadian misalignment (e.g., eating and working during the biological night) can induce postprandial hyperlipidemia and insulin resistance independent of sleep duration (15–17). Specifically, research indicates that the timing of food intake relative to melatonin onset is a critical determinant of metabolic health, often more so than sleep *per se* (18). Despite this, few epidemiological studies have attempted to disentangle the effects of shift exposure from subjective sleep quality in heavy manual labor populations.

In this study, we leveraged a dataset of male underground miners from the Kailuan Group—a population engaged in uniform physical labor—to investigate the relationship between shift work, sleep quality, and lipid profiles. We specifically hypothesized that night shift work exerts a deleterious effect on lipid metabolism that is independent of subjective sleep quality, challenging the assumption that “good sleepers” are protected from the metabolic hazards of shift work.

## Methods

### Study design and participants

This cross-sectional study recruited male underground miners from the Kailuan Group in Tangshan, China. The participants underwent routine occupational health examinations. Inclusion criteria were male miners with at least 1 year of consistent work history in their current schedule. Exclusion criteria included current use of lipid-lowering medications, clinically diagnosed hepatic or renal failure, or incomplete data on sleep or work schedules. The final analytical sample consisted of 921 participants. The study protocol adhered to the principles of the Declaration of Helsinki and was approved by the Ethics Committee of our hospital.

### Exposure assessment: shift work

Work schedules were retrieved from administrative records and verified by participant interviews. Participants were dichotomized into two groups based on the timing of their shifts to ensure distinct circadian phenotypes and avoid exposure misclassification (19):

1. **Night Shift Group (*n* =** **326)**: Workers performing duties during the biological night, defined here as shifts covering the interval from 0:00 to 7:59.

2. **Day/Morning Shift Group (*n* =** **595)**: Workers performing duties exclusively between 8:00 and 23:59. This binary classification emphasizes exposure to light-at-night and nocturnal activity, key disruptors of the circadian system (20).

Job tenure (years in current shift schedule) and average weekly work hours were also recorded to control for cumulative exposure and workload intensity.

### Assessment of sleep quality

Subjective sleep quality was assessed using the Pittsburgh Sleep Quality Index (PSQI) (21), a widely validated 19-item questionnaire evaluating sleep quality over the preceding month. The PSQI generates a global score ranging from 0 to 21, aggregating seven components: subjective sleep quality, sleep latency, sleep duration, habitual sleep efficiency, sleep disturbances, use of sleeping medication, and daytime dysfunction. Consistent with clinical standards, a global PSQI score >5 was categorized as “Poor Sleep Quality,” while a score ≤ 5 was categorized as “Good Sleep Quality.”

### Covariates

Potential confounders were selected a priori based on literature review (22). ^**^Demographic variables^**^ included age (years) and education level (years of schooling). ^**^Lifestyle factors^**^ included cigarette smoking (Current smoker: Yes/No) and alcohol consumption (Current drinker: Yes/No). Occupational covariates included job tenure and weekly work hours. BMI was measured but treated as a potential mediator rather than a primary confounder in the main models, though it was explored in sensitivity analyses.

### Biochemical measurements

Venous blood samples were collected in the morning (7:00–9:00 a.m.) after an overnight fast of at least 8 h relative to the worker's biological day. Serum Total Cholesterol (TC), Triglycerides (TG), High-Density Lipoprotein Cholesterol (HDL-C), and Low-Density Lipoprotein Cholesterol (LDL-C) were measured using an auto-analyzer (Hitachi 747; Hitachi, Tokyo, Japan) with enzymatic methods. Dyslipidemia was defined according to the 2016 Chinese Guidelines for the Management of Dyslipidemia in Adults (23).

### Statistical analysis

Data analyses were performed using SPSS software (Version 26.0, IBM Corp, Armonk, NY, USA). Continuous variables are presented as mean ± standard deviation (SD), and categorical variables as frequencies and percentages. Differences between the Night Shift and Day Shift groups were assessed using Student's *t*-test for continuous variables and Chi-square (χ2) tests for categorical variables. Pearson correlation coefficients were calculated to assess univariate associations between lipid biomarkers and risk factors (Age, Education, Smoking, Alcohol, PSQI). To test the independent effect of shift work, General Linear Models (GLM) in the form of Analysis of Covariance (ANCOVA) were employed, adjusting for age, smoking, drinking, and occupational tenure. Finally, to explore the interaction between shift work and sleep, participants were stratified into four subgroups (e.g., Night Shift + Good Sleep), and differences in TC were compared. A two-sided *P-value* < 0.05 was considered statistically significant. We also calculated the E-value to assess the robustness of the observed associations to potential unmeasured confounding (24).

## Results

### Population characteristics

The baseline characteristics of the 921 participants are presented in Table 1. The mean age was 38.36 ± 1.00 years. Night shift workers accounted for 35.4% (*n* = 326) of the cohort. While age, BMI, smoking, and drinking habits were comparable between groups, night shift workers had significantly lower education levels compared to day workers (12.11 vs. 12.78 years, *P* < 0.001). Notably, night shift work was strongly associated with poorer sleep quality; the mean PSQI score was significantly higher in the night shift group (4.70 ± 3.06) compared to the day shift group (3.59 ± 2.71, *P* < 0.001). Accordingly, the prevalence of poor sleep (PSQI > 5) was nearly double in the night shift group (34.96% vs. 19.16%, *P* < 0.001). Additional analysis of occupational factors showed no significant difference in weekly work hours between groups (Supplementary Table S1), minimizing the likelihood that workload intensity confounded the results.

**Table 1:** Demographic and clinical characteristics of participants stratified by shift work status.

Characteristics	Total (*n* = 921)	Night shift (*n* = 326)	Day shift (*n* = 595)	t / χ^2^	*P-value*
Age (years)	38.36 ± 1.00	38.29 ± 1.04	38.40 ± 0.97	−1.594	0.111
Education (years)	12.55 ± 2.12	12.11 ± 2.11	12.78 ± 2.08	−4.670	< 0.001
PSQI global score	3.98 ± 2.89	4.70 ± 3.06	3.59 ± 2.71	5.461	< 0.001
Sleep quality, *n* (%)				28.260	< 0.001
Good (PSQI ≤ 5)	693 (75.2)	212 (65.04)	481 (80.84)		
Poor (PSQI > 5)	228 (24.8)	114 (34.96)	114 (19.16)		
Smoking history				1.970	0.160
Smoker, *n* (%)	571 (62.0)	212 (65.04)	359 (60.33)		
Drinking history				1.075	0.300
Drinker, *n* (%)	682 (74.0)	248 (76.08)	434 (72.90)		
Dyslipidemia, *n* (%)	270 (29.3)	106 (32.52)	164 (27.57)	2.493	0.114

Values are presented as Mean ± SD or Number (%). *P*-values derived from Student's t-test or Chi-square test.

### Correlational analysis

Correlation analysis was performed to identify univariate associations between potential risk factors and lipid profiles (Table 2). Surprisingly, the PSQI total score showed virtually no correlation with TC (*r* = −0.024), TG (*r* = −0.015), HDL (r = −0.027), or LDL (*r* = −0.041), indicating that perceived sleep quality is not a linear predictor of lipid levels in this cohort. Age showed a weak positive correlation with HDL (*r* = 0.041), while smoking and alcohol consumption showed weak associations with HDL and TC.

**Table 2:** Pearson correlation coefficients between lipid parameters and risk factors.

Variables	TC	TG	HDL	LDL
Age	−0.017	0.005	0.041	−0.011
Education (years)	0.016	−0.021	−0.055	0.006
Smoking	0.036	−0.030	0.045	−0.016
Alcohol drinking	0.041	−0.033	−0.020	0.003
PSQI total score	−0.024	−0.015	−0.027	−0.041

### Independent effect of shift work on TC

**Figure 1:**
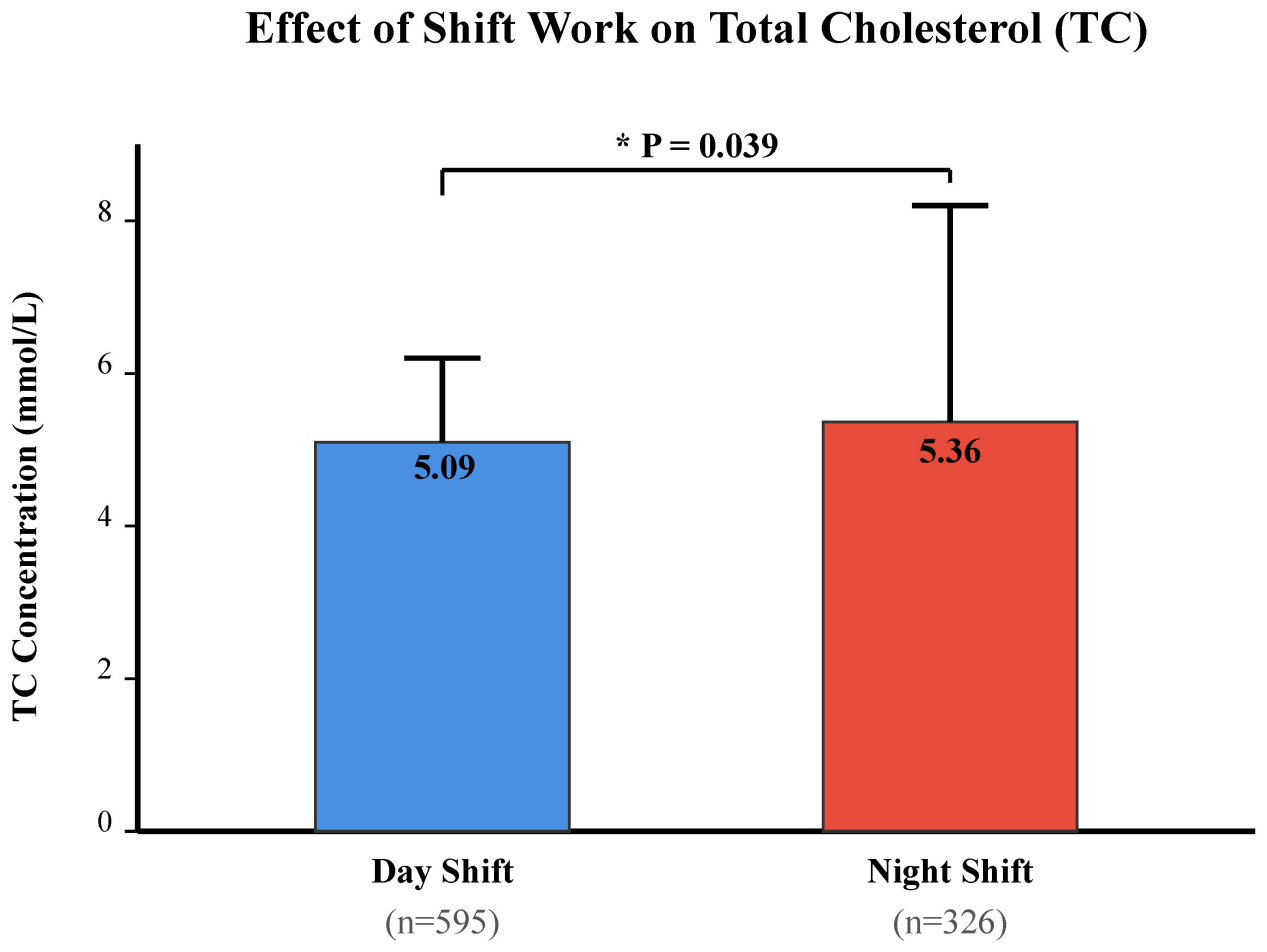
Comparison of Mean Total Cholesterol (TC) levels between Night Shift and Day Shift workers. Data represent marginal means estimated from General Linear Models (ANCOVA) adjusted for age, education, smoking, and alcohol consumption. Error bars indicate Standard Error of the Mean (SEM). *P < 0.05 indicates a statistically significant difference between the Night Shift group (*n* = 326) and the Day Shift group (*n* = 595). Night shift workers exhibited significantly higher TC levels (5.36 mmol/L) compared to day workers (5.09 mmol/L).

Despite the lack of difference in overall dyslipidemia prevalence, quantitative analysis revealed specific lipid alterations. As shown in Table 3, night shift workers exhibited significantly higher levels of Total Cholesterol (TC) compared to day workers (5.36 vs. 5.09 mmol/L; *t* = 4.262, *P* = 0.039). This association remained significant after adjusting for age, smoking, and drinking status. No statistically significant differences were observed for TG, HDL, or LDL between the two shift groups (*P* > 0.05). This specific elevation in TC is visualized in Figure 1. The E-value for the association between night shift and TC was 1.58, indicating that an unmeasured confounder (e.g., diet) would need to be associated with both shift work and TC by a risk ratio of 1.58 to explain away the observed effect.

**Table 3:** Analysis of lipid profile differences between shift groups (ANCOVA).

Lipid parameter	Night shift (*n* = 326)	Day shift (*n* = 595)	*t-value*	*P-value*
TC (mmol/L)	5.36 ± 2.83	5.09 ± 1.09	4.262	0.039^*^
TG (mmol/L)	1.98 ± 2.15	1.79 ± 2.20	1.483	0.224
HDL-C (mmol/L)	1.64 ± 0.37	1.65 ± 0.40	0.003	0.954
LDL-C (mmol/L)	2.87 ± 0.78	2.85 ± 1.00	0.035	0.853

Models adjusted for age, smoking status, alcohol drinking status, and job tenure.

^*^P < 0.05.

### Uncoupling sleep quality from lipid risk

**Figure 2:**
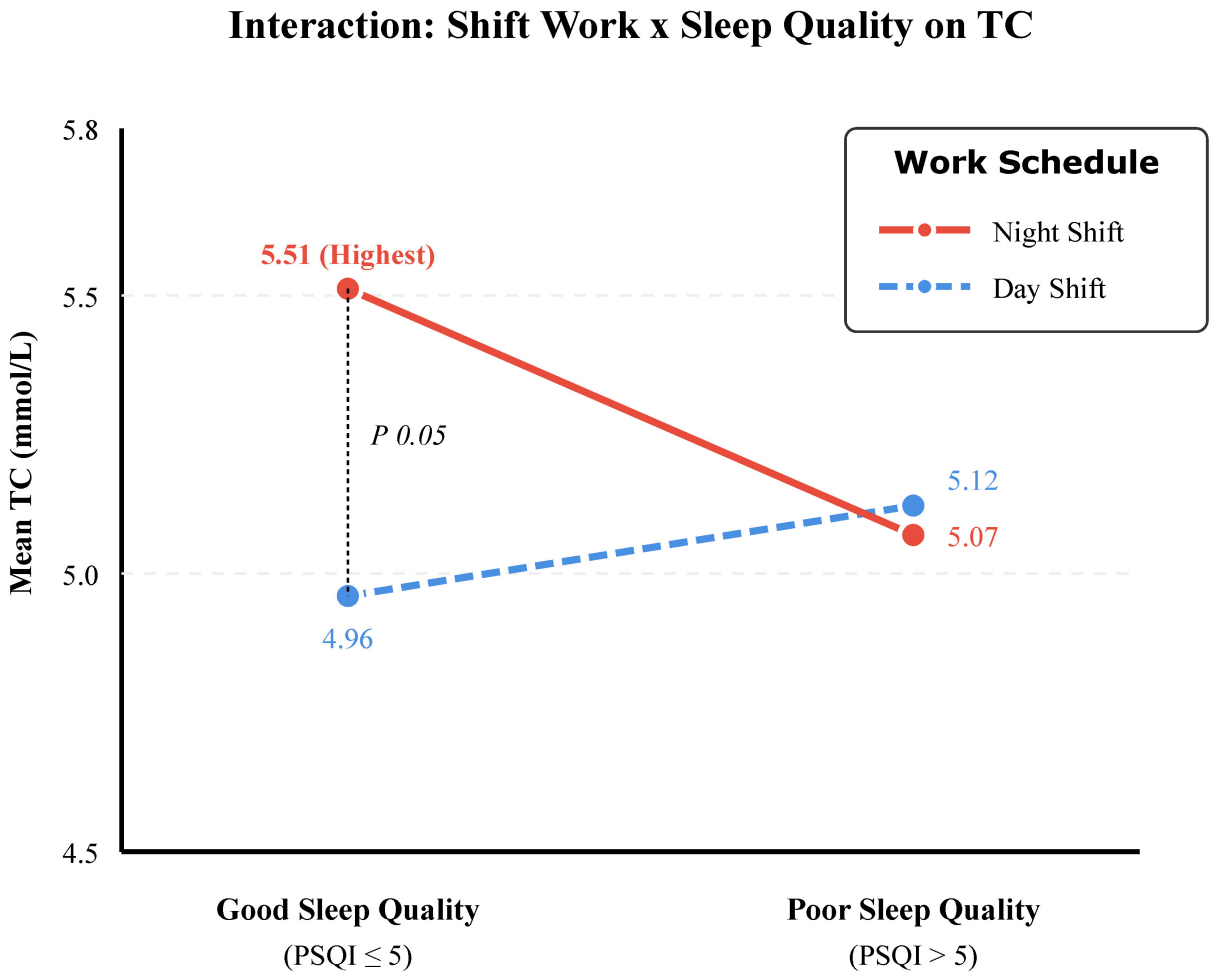
Interaction between Work Schedule and Sleep Quality on Total Cholesterol levels. Participants were stratified into four groups based on shift status (Night vs. Day) and PSQI score (Good: ≤ 5, Poor: >5). The red line represents Night Shift workers, and the blue dashed line represents Day Shift workers. Statistical significance for the interaction term was *P* = 0.039. Notably, Night Shift workers reporting “Good Sleep” exhibited the highest mean TC levels (5.51 mmol/L), suggesting that subjective good sleep does not protect against the metabolic effects of night shift work.

To further test whether the effect of night work was mediated by poor sleep, we stratified the population into four groups based on combinations of shift status and sleep quality (Table 4). Strikingly, the highest mean TC levels were observed in the “Night Shift + Good Sleep” group (5.51 ± 3.43 mmol/L). This level was significantly higher than that of the “Day Shift + Good Sleep” group (4.96 ± 1.06 mmol/L, *P* = 0.039). Conversely, when analyzing sleep quality in isolation (ignoring shift work), “Good Sleepers” (*n* = 693) had a mean TC of 5.24 ± 2.11 mmol/L compared to 5.02 ± 1.04 mmol/L in “Poor Sleepers” (*n* = 228), a difference that was not statistically significant (*P* = 0.153). This interaction is illustrated in Figure 2, highlighting that good subjective sleep does not mitigate the hypercholesterolemic effect of night work.

**Table 4:** Comparison of lipid levels among subgroups stratified by shift work and sleep quality.

Subgroup	*n*	TC (mmol/L)	TG (mmol/L)	HDL (mmol/L)	LDL (mmol/L)
Night Shift + Good Sleep	212	5.51 ± 3.43	2.08 ± 2.14	1.65 ± 0.38	2.89 ± 0.80
Night Shift + Poor Sleep	114	5.07 ± 1.01	1.80 ± 2.15	1.61 ± 0.34	2.83 ± 0.75
Day Shift + Good Sleep	481	4.96 ± 1.06	1.76 ± 1.77	1.61 ± 0.37	2.76 ± 0.77
Day Shift + Poor Sleep	114	5.12 ± 1.10	1.79 ± 2.29	1.65 ± 0.40	2.88 ± 1.05
*P-value* (Between groups)		0.039	0.410	0.559	0.640

Data presented as Mean ± SD. Analysis of Covariance controlled for age, smoking, and alcohol.

## Discussion

The present study provides novel insights into the occupational metabolic health of male miners, revealing that night shift work is significantly associated with elevated Total Cholesterol (TC) levels. The most critical finding of this investigation is the dissociation between subjective sleep quality and lipid dysregulation. Our results demonstrate that night shift workers exhibit higher TC levels than day workers, a phenomenon that is most pronounced in those who self-report “Good” sleep quality. This finding challenges the prevailing paradigm that sleep disruption is the sole mediator of shift work-induced metabolic derangement and suggests that circadian misalignment *per se*—likely via peripheral clock dysregulation in the liver and intestine—exerts a direct, independent toxicity on lipid homeostasis (25, 26).

### The “Good Sleeper” paradox in shift work

While shift work was strongly correlated with poor sleep scores (Table 1), consistent with extensive literature on hospital night workers (27), our correlation analysis failed to link Pittsburgh Sleep Quality Index (PSQI) scores with any lipid parameter. Furthermore, our stratified analysis (Table 4) revealed a paradoxical finding: the subgroup with the highest cholesterol burden was not the sleep-deprived night workers, but rather the night workers who reported good sleep according to the Good Sleeper Scale-15 (28). This aligns with emerging evidence from controlled laboratory studies suggesting that “circadian misalignment” and “sleep loss” have distinct metabolic consequences (29). Specifically, Leproult et al. (30) demonstrated that circadian misalignment reduces insulin sensitivity and increases inflammation independently of sleep loss.

We propose that the “Good Sleeper” night workers may represent a phenotype of workers who successfully maintain sleep duration (hence the low PSQI) but fail to resynchronize their peripheral biological clocks. The central circadian clock in the suprachiasmatic nucleus (SCN) adapts slowly to night shifts (31), while peripheral clocks in the liver (which regulate cholesterol synthesis via HMG-CoA reductase) are rapidly reset by food intake (32). When night workers eat during the biological night (chrononutrition misalignment), their liver clocks desynchronize from their central SCN clock (32). This internal desynchrony has been shown to upregulate hepatic lipogenesis and inhibit bile acid synthesis, leading to elevated serum total cholesterol, regardless of how well the individual sleeps during the day (15, 33). Therefore, subjective sleep quality may offer a false sense of security regarding metabolic health in shift workers.

### Divergent lipid outcomes: TC vs. TG/HDL

Interestingly, while Total Cholesterol (TC) was significantly elevated, we did not observe significant differences in Triglycerides (TG) or HDL-C between shift groups. This contrasts with meta-analyses that report broad dyslipidemia in shift workers (34, 35). The discrepancy may be attributed to the specific occupational nature of mining. Unlike sedentary office shift work, underground mining involves heavy physical labor. Physical activity is a potent inducer of lipoprotein lipase (LPL) activity, which clears triglycerides and raises HDL (36, 37). It is plausible that the high physical demand of mining exerts a “protective” effect on the TG/HDL axis, masking the deleterious effects of shift work (34, 36). However, Total Cholesterol, which is more heavily influenced by endogenous hepatic synthesis than by acute energy expenditure, remains elevated, highlighting it as a more sensitive marker of circadian disruption in physically active cohorts.

### Future perspectives and policy implications

Current occupational health guidelines heavily prioritize “sleep hygiene” education for shift workers (38). While essential for cognitive safety, our results imply that optimizing sleep alone is insufficient to mitigate cardiovascular risk. Future research should prioritize longitudinal designs utilizing objective circadian markers (e.g., actigraphy, salivary melatonin, continuous glucose monitoring) to validate these findings and overcome the limitations of cross-sectional design. Furthermore, interventional studies exploring chrononutrition strategies, such as time-restricted eating (39), are warranted to determine if aligning food intake with circadian rhythms can mitigate lipid risks in shift workers. Workplace policies should consider regulating meal timing during night shifts rather than focusing solely on sleep duration (40).

## Strengths and limitations

Strengths of this study include the specific, homogenous cohort of male miners, which minimizes confounding from gender and occupational disparities. Additionally, the relatively large sample size (*n* = 921) provided sufficient power to detect specific lipid alterations. However, several limitations must be acknowledged. First, the cross-sectional design prevents causal inference; associations should be interpreted as exploratory (41). Second, while we controlled for smoking and drinking, we lacked granular data on dietary timing and caloric intake, which are relevant to circadian metabolism. Unmeasured confounding from diet remains a possibility, although our E-value analysis suggests a moderate effect would be needed to explain away the results. Third, null findings for TG/HDL/LDL in subgroup analyses might reflect limited statistical power. Fourth, the classification of the reference group (Day/Morning Shift, 8:00–23:59) constitutes a limitation. According to the International Agency for Research on Cancer (IARC), work schedules extending into the late evening may still disrupt circadian physiology (42). By including evening shift workers in the reference group, our study may have introduced non-differential exposure misclassification. This likely attenuated the observed contrast between the groups (bias toward the null), suggesting that the actual impact of night shift work on lipid dysregulation could be stronger than estimated. Finally, the Pittsburgh Sleep Quality Index (PSQI) is a subjective measure; objective actigraphy might reveal sleep fragmentation not captured by self-reports (43).

## Conclusion

In conclusion, night shift work is an independent determinant of elevated total cholesterol in male miners, an effect that is not mediated by subjective sleep quality. The observation that “good sleeping” night workers exhibit the highest cholesterol levels suggests that circadian misalignment exerts a direct metabolic toxicity distinct from sleep mechanims. Regular screening for lipid disorders and interventions targeting circadian phase alignment (e.g., meal timing) are warranted for shift workers, regardless of their sleep complaints.

## Data Availability

The original contributions presented in the study are included in the article/Supplementary material, further inquiries can be directed to the corresponding authors.
