# Supplementary material for: Dissociation between subjective sleep quality and lipid dysregulation in underground miners: night shift work as an independent risk factor for hypercholesterolemia
Source: Front Public Health. 2026 Apr 13;14:1759787. doi: 10.3389/fpubh.2026.1759787 (PMC13111404; doi:10.3389/fpubh.2026.1759787)
Supplement: Supplementary file 1 [file Table_1.docx]

**Supplementary Table S1.** Comparison of Occupational factors between groups

| **Characteristic** | **Night Shift (n=326)** | **Day Shift (n=595)** | **t-value** | **P-value** |
| --- | --- | --- | --- | --- |
| Job Tenure (years) | 14.2 ± 5.1 | 14.5 ± 4.9 | -0.85 | 0.395 |
| Weekly Work Hours | 48.5 ± 4.2 | 47.9 ± 3.8 | 1.92 | 0.055 |
